# Screening for Hypophosphatasia in Adult Patients at a Maximum Care Provider—Retrospective Analyses over Fifteen Years

**DOI:** 10.3390/jcm13237313

**Published:** 2024-12-02

**Authors:** Robert Hennings, Diana Le Duc, Linnaeus Bundalian, Anke Tönjes, Johannes R. Lemke, Joachim Thiery, Jürgen Kratzsch, Andreas Roth

**Affiliations:** 1 Department of Orthopaedic Surgery, Traumatology and Plastic Surgery, University Hospital Leipzig, Liebigstrasse 20, 04103 Leipzig, Germany; andreas.roth@medizin.uni-leipzig.de; 2 Department of Human Genetics, Faculty of Medicine, University of Leipzig, 04103 Leipzig, Germany; gabriela-diana.leduc@medizin.uni-leipzig.de (D.L.D.); linnaeus.bundalian@medizin.uni-leipzig.de (L.B.);; 3 Medical Department III—Endocrinology, Nephrology, Rheumatology, University of Leipzig Medical Center, Liebigstrasse 20, 04103 Leipzig, Germany; anke.toenjes@medizin.uni-leipzig.de; 4 Medical Faculty, University of Kiel, CAU Kiel, 24105 Kiel, Germany; dekan@uksh.de; 5 Institute of Laboratory Medicine, Clinical Chemistry and Molecular Diagnostics, University of Leipzig, 04109 Leipzig, Germany; juergen.kratzsch@medizin.uni-leipzig.de

**Keywords:** alkaline phosphatase, hypophosphatasia, hypophosphatasemia, *ALPL gene*, *ALPL gene* variant, pathogenic nucleotide variants

## Abstract

**Background/Objectives:** Hypophosphatasemia (HPE) may be temporary (tHPE) in the context of severe diseases, such as sepsis or trauma, or it may persist (pHPE), indicating an adult form of hypophosphatasia (HPP; OMIM 171760), a rare metabolic bone disorder caused by pathogenic nucleotide variants (PNVs) in the *ALPL* ***gene***. The aim of this study was to analyze the role of auxiliary general biomarkers in verifying low alkaline phosphatase (ALP) serum activity level as an alert parameter for PNVs in the *ALPL* ***gene***, which are indicative of HPP. In this retrospective analysis, we examined adult patients with an ALP serum activity level below 21 U/L. The cohort comprised 88 patients with temporary HPE (tHPE group) and 20 patients with persistent HPE who underwent re-examination. Genetic analysis performed on 12 pHPE patients identified PNV in the *ALPL* ***gene*** in 11 cases (ALPL group). Hemoglobin [HB], aspartate aminotransferase [AST], gamma-glutamyl transferase [GGT], calcium, phosphate, thyrotropin [TSH], albumin, total protein, and C-reactive protein [CRP] levels represented basic biomarkers. A comparative analysis between groups employed a Student’s *t*-test, and a Student’s *t*-test with bootstrap sampling (*n* = 10.000) was performed. **Results:** The mean HB, ALP, calcium, albumin, and total protein levels were lower in the tHPE group compared with the ALPL group (*p* < 0.01). AST and CRP were increased in the tHPE group (*p* < 0.01). The model showed an accuracy of 90% and an AUC of 0.94, which means that it can discern the two groups ~94% of the time. **Conclusions:** Basic biomarker evaluation effectively supports the interpretation of a decreased ALP serum activity level in the context of suspected HPP. In patients with laboratory HPE and biomarkers within reference, a PNV in the *ALPL* ***gene*** is highly suspected.

## 1. Introduction

Adult hypophosphatasia (HPP; OMIM 171760) is a rare inherited disorder characterized by defective bone mineralization and a deficiency of tissue-nonspecific alkaline phosphatase (TNSALP) activity [1,2,3,4,5]. This inherited disorder is caused by loss-of-function mutations in the *ALPL **gene*** (MIM 171760) on chromosome 1p36.1-p34. More than 440 pathogenic nucleotide variants (PNVs) in the *ALPL **gene*** (including missense, nonsense, splice site, and frameshift deletions and insertions) have been described in the *ALPL **gene*** Variant Database of the Johannes Kepler University (JKU) [6,7]. The prevalence of severe forms of HPP varies between 0.3 and 1/100,000. In model calculations, a prevalence of 1/6370 regarding mild forms could be determined [4]. The prevalence of severe forms varies between 0.3 and 1/100,000. The diagnosis of HPP can be challenging due to its unspecific clinical presentation, with musculoskeletal pain being a common symptom in adult patients [8,9,10,11]. Decreased alkaline phosphatase (ALP) serum activity level is often the first indicator leading to a suspicion of HPP [12].

But ALP serum activity levels measured by commercially available test systems include all isoforms of the enzyme. Up to 90% of ALP serum activity is generated by tissue-nonspecific alkaline phosphatases (TNSALPs) from the liver and bone, each contributing 50% [5,13,14]. The remaining 10% is caused by tissue-specific ALPs, e.g., from placental, intestinal, and germ line cells [13,15]. It should be noted that temporary low ALP serum activities levels, known as hypophosphatasemia (HPE), can be induced by hypomagnesemia and hypocalcemia. Additionally, HPE is a consequence of major surgery, severe injury, multiple myeloma, massive blood transfusion, heart diseases, or infections [3,16]. Thus, differentiating temporary from persistent HPE as an indicator of HPP is of clinical importance. In addition, further HPP-specific biomarkers can be measured, including bone-specific alkaline phosphatase (BALP) and the substrate of TNSAP, pyridoxal-5′-phosphate (PLP) [12]. If clinical and laboratory findings are unclear, a genetic analysis is indicated, but is not mandatory [17]. Taken together, HPP-specific diagnosis is expensive, as well as time-consuming and not available at all facilities.

Adult patients with HPP require comprehensive management that includes assessing bone and joint complications, chronic pain, and mood disorders [18,19]. The adult form of HPP can be accompanied by insufficiency fractures, in particular, of the metatarsalgia, subtrochanteric femur fractures, chondrocalcinosis, periodontal diseases, and unspecific complaints of the musculoskeletal system like muscle weakness [8,9,19,20]. Diagnostic delays are common in HPP, emphasizing the need for increased awareness and timely identification of the condition [21]. The Global HPP Registry data indicate that the median time between the onset of symptoms and the diagnosis of HPP is 5.7 years [22]. Atypical femoral fractures have been reported in adult patients with hypophosphatasia, highlighting the importance of considering this condition in cases of unusual fractures, especially during bisphosphonate exposure [23,24]. Furthermore, the use of asfotase alfa has shown success in treating adult patients with childhood-onset HPP, indicating the potential for enzyme replacement therapy in managing this condition [17,25,26]. Asfotase alfa is a tissue-nonspecific, human recombinant alkaline phosphatase Fc-deca-asparaginate that replaces the natural form. It is administered as an orphan drug subcutaneously in a weight-adapted manner [27]. In this paper, we present a retrospective analysis spanning fifteen years and focusing on screening for HPP in adult patients at a maximum care provider. The objective of this study was to assess the diagnostic potential of commonly available biomarkers associated with tHPE and pHPE in relation to the potential pathogenic nucleotide variants (PNVs) in the *ALPL **gene***, which are indicative of HPP. Based on the findings, a laboratory algorithm should be presented that enables early assessment of decreased ALP serum activity levels in the context of hypophosphatasia (HPP).

## 2. Materials and Methods

### 2.1. Patients and Study Groups

This retrospective study was approved by the local ethics committee (no. 508/16-ek). The study was conducted in accordance with the principles of the Declaration of Helsinki and in accordance with the guidelines for Good Clinical Practice. All participants provided written consent for the use of their anonymized data in the treatment contract. Patients who underwent follow-up procedures provided separate written consent for participation and for genetic analysis.

All laboratory data of adult patients (older than 18 years) of the University Hospital Leipzig (UHL) for the years 2000–2015 were retrospectively analyzed to identify patients with ALP serum activity levels ≤ 21 U/L. The study cut-off of 21 U/L was based on reports from adult patients with confirmed clinical manifestations of severe HPP [23]. Hemoglobin (HB), aspartate aminotransferase (AST), gamma glutamyl transferase (GGT), calcium, phosphate, thyrotropin (TSH), albumin, total protein, and C-reactive protein (CRP) levels were defined as general biomarkers and measured at baseline and at follow-up.

All ALP serum activity levels available at the UHL were analyzed. The reference range was 35–105 U/L for healthy women and 40–130 U/L for healthy men [19,20]. A total of 204 patients with at least one measured ALP serum activity levels < 21 U/L were selected [18]. In cases of multiple ALP serum activity levels, the lowest was used as the inclusion value. After analysis of their medical history, 134 (66%) of the 204 patients had physiological ALP serum activity levels as defined by tHPE. Of these, 46 had no measurement of other common biomarkers and were therefore excluded. In 88 of them, at least one of the general biomarkers was measured simultaneously. They were included in the temporary hypophosphatasemia group (*n* = 88, tHPE group).

**Figure 1:**
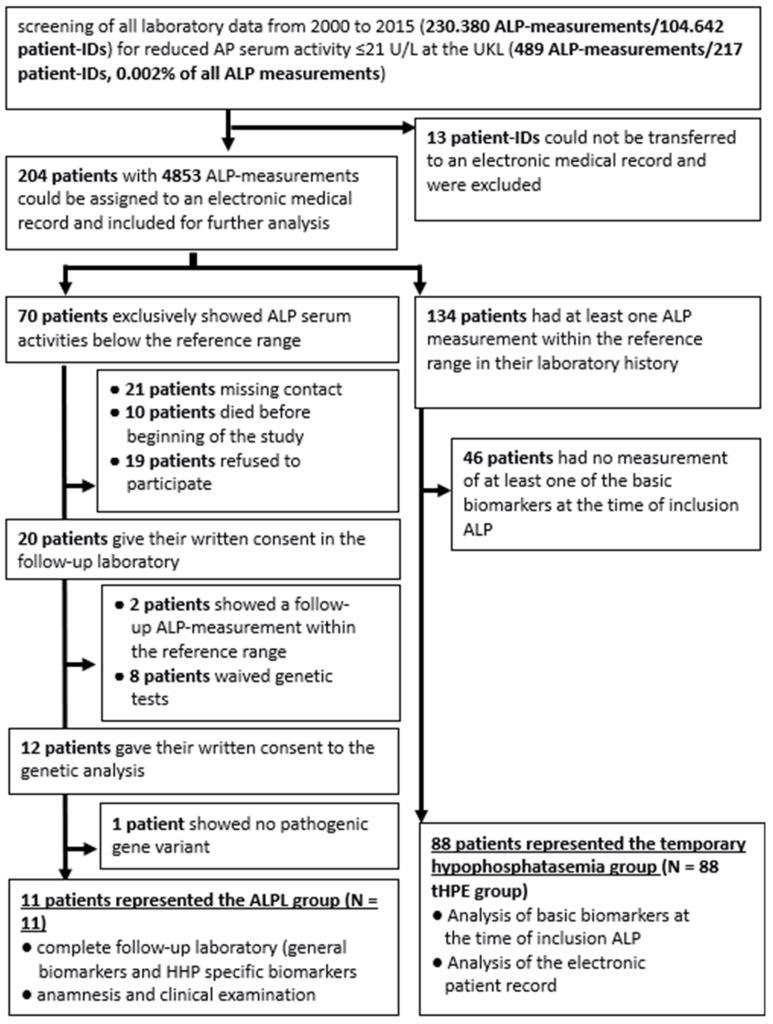
Flowchart of the assessment of the patients and the resulting study groups. UHL = University Hospital of Leipzig.

A total of 20 of the remaining 70 patients with solely decreased ALP serum activity levels were contacted and agreed to participate in a follow-up examination, including a medical history and a follow-up laboratory test (Figure 1). In total, 18 of these showed a decrease in ALP serum activity level at follow-up and have been further investigated. Two had ALP serum activity levels within reference ranges at follow-up and were excluded.

### 2.2. Methods

Blood samples were obtained between 8:00 and 10:00 a.m. after a 12 h fast. Two S-Monovettes^®^ 2.7 mL K3EDTA and one S-Monovette^®^ 4.7 mL serum (Sarstedt AG & Co. KG, Postfach 1220, D-51588 Nümbrecht, Germany) were used. All biomarkers were measured at the Institute of Laboratory Medicine, Clinical Chemistry and Molecular Diagnostics (ILM), UHL, using commercially certified biochemical assays. The determination of ALP activity in serum was performed using the “Alkaline Phosphatase according to IFCC Gen.2 ALP2” kit (Roche Diagnostics Deutschland GmbH, 68305 Mannheim, Germany) on a cobas^®^ c501 instrument using the release of p-nitrophenol after cleavage of p-nitrophenyl phosphate by the enzyme. The product can be quantified by extinction.

Specific bone alkaline phosphatase (BALP) and serum level of its substrate, pyridoxal-5′-phosphate, were analyzed as HPP-specific biomarkers and are generally accepted for this purpose [5,8].

The Ostase^®^ BAP EIA assay (Immunodiagnostic Systems Holdings, Boldon Colliery, United Kingdom) was used to quantify BALP. BALP was purified by immunosorbent assay (EIA) using biotin-linked monoclonal antibodies and quantified using the same enzyme assay described for ALP serum activity levels.

PLP levels were measured using vitamin B6 in Serum/Plasma HPLC kit (Chromsystems Instruments & Chemical, Gräfelfing, Germany). After protein precipitation, vitamin B6 was derivatized and separated by HPLC. Quantitation was performed by fluorescence (415 nm).

Genetic analysis was performed at the Institute of Human Genetics at the UHL by targeted enrichment of the gene target regions of the human exome plus adjacent intronic regions (±10 bp) using the TruSight^®^One enrichment kit (Illumina Inc., San Diego, CA, USA). More than 95% of the target regions were covered at least twenty-fold. Sequencing was performed on a NextSeq 550 sequencing instrument (Illumina Inc., San Diego, CA, USA) using 150 base-pair paired-end reads. Varvis software (Limbus Medical Technologies GmbH, Rostock, Germany) was used for bioinformatic analysis.

### 2.3. Statistics

Statistical analysis was performed using SPSS (version 24, IBM) for Windows (Microsoft). Independent samples with normal distribution were calculated using the two-tailed *t*-test, and frequency differences were calculated using the chi-squared test.

Furthermore, a Student’s *t*-test with bootstrap sampling method was performed to test the groups and evaluate whether the ALPL group had significantly lower values for a biomarker compared to the tHPE group. The comparison was made between groups derived from the bootstrap sampling for each iteration (*n* = 10,000), where we randomly picked a subset of the majority group to address class imbalance. We developed two logistic regression models to determine which will be a better predictor for the ALPL or tHPE, using alkaline phosphatase biomarker (U/L) or multiple biomarkers: alkaline phosphatase (U/L), aspartate aminotransferase (µkat/L), gamma-glutamyl transferase (µkat/L), hemoglobin (mmol/L), creatinine (µmol/L), calcium (mmol/L), thyrotropin (mU/L), C-reactive protein (mg/L), albumin (g/L), and total protein (g/L) were developed.

## 3. Results

### 3.1. Comparison of tHPE Group and ALPL Group

Within and between the tHPE and ALPL groups, the sex and age distributions were statistically comparable (Table 1; *p* > 0.05). The frequencies of pathological findings for the general biomarkers were equally distributed between the sexes in both the tHPE and ALPL groups (*p* > 0.05). Therefore, sex-specific analysis was not performed. Pathological values for HB, ALT, AST, creatine, calcium, albumin, total protein, and CRP occurred significantly more frequently in the tHPE group than in the ALPL group (*p* ≤ 0.05; Table 2). The mean levels of HB, ALP, calcium, albumin, and total protein were lower in the tHPE group than in the ALPL group (*p* < 0.05; Table 2). However, analysis of the serum levels of CRP and AST showed significantly higher values in the tHPE group (*p* ≤ 0.05). No significant differences were found between the tHPE and ALPL groups for creatinine, GGT, and TSH levels (*p* > 0.05; Table 2).

In contrast to the ALPL group, all patients in the tHPE group had known additional diseases independent of HPP that are typical of tHPE, such as severe anemia, sepsis, and gastrointestinal, hematological, and rheumatological diseases (Table 1).

### 3.2. Patients of the ALPL Group

All patients in the ALPL group (N = 11) exhibited a heterozygous PNV in the *ALPL **gene***. Eight distinct PNVs were identified, each associated with pathological values in the HPP-specific laboratory. The following PNVs were detected: c.119C > T; c.1250A > G; c.1171del; c.1331A > G; c.406C > T; c.341C > T; c.1190-2A > T; c.297G > A. Patient characteristics are detailed in Table 3. Furthermore, all patients in the ALPL group exhibited clinical symptoms consistent with suspected HPP (100% exhibited recurrent musculoskeletal complaints, 27% exhibited premature caries, and a 9% prevalence of premature loss of primary teeth). At the start of the study, none of the patients in the ALPL group had a known history of HPP or permanent ALP reduction.

In the ALPL group, HB, ALT, calcium, albumin, total protein, and CRP were within reference ranges in all follow-up laboratory data. Three patients (27%) had elevated GGT levels, two (18%) had elevated phosphate levels, one (9%) had decreased phosphate levels, one (9%) had elevated TSH or AST levels, and one (9%) had elevated creatinine levels at follow-up. All patients in this group had decreased BALP levels and/or increased PLP levels.

### 3.3. Logistic Regression Models

The first and second model have the same accuracy of 90% (Table 4). Even though that is the case, the second model has a higher AUC value (AUC = 0.94), which implies that it can discern the two groups ~94% of the time. The coefficient values for each biomarker, presented in Table 5, reflect an increase or decrease in the likelihood of change in the group assignment per unit change, depending on the direction of the coefficient, thereby elucidating each variable’s impact on group classification.

## 4. Discussion

A persistent reduction in ALP serum activity level is a hallmark feature of HPP [19]. This is a crucial factor in differentiating HPP from other diseases such as rickets [28]. In clinical practice, it is essential to pay close attention to borderline or marginally reduced ALP serum activity values, as these may be indicative of HPP and could otherwise be overlooked [11].

The causes of low ALP serum activity levels are numerous and vary according to the tHPE [3,10]. The prevalence of hypophosphatemia of any cause is reported to be 1.18% to 8.46% [10,29]. In the author’s opinion, first, it is essential that the assessment of a reduced ALP serum activity level takes into account other serum parameters that could represent differential diagnoses. This study shows that HB, AST, calcium, albumin, total protein, and CRP were significantly more often in the physiological range in patients carrying a heterozygous PNV in the *ALPL* ***gene*** than in patients with tHPE. Thus, if the serum activity level of ALP is decreased and these other values are within the reference range, a PNV in the *ALPL **gene*** is highly suspected in these cases, which may be a sign of possible HPP. On the other hand, if ALP serum activity level is decreased, and the other values are in the pathological range, a differential diagnosis other than HPP should be considered. The results presented here are consistent with the laboratory and clinical findings reported by Larid et al. and represent a valuable contribution to the existing body of knowledge [10,19].

To our knowledge, this is the first study to present general biomarkers that can be used for the first-line assessment of reduced ALP activity in relation to possible HPP-causing PNVs in the *ALPL **gene*** in adults.

### 4.1. Differential Diagnosis of HPE

The results of the present study align with those of previous research, which suggest that a single reduction in ALP activity does not necessarily indicate the presence of HPP. The proportion of patients with tHPE was shown to be between 66.7% and 72.4%, which is consistent with the demonstrated 66% [10,30,31]. There are numerous reasons for transient HPE. A study conducted by Lum et al. (1995) revealed that in 47% of patients with tHPE, other medical conditions accounted for the observed decrease in ALP serum activity levels [10,16]. These included cardiac surgery and cardiopulmonary bypass (26.5%), malnutrition (12.0%), magnesium deficiency (4.8%), hypothyroidism (2.4%), and severe anemia (1.2%). A total of 53% of cases exhibited no identifiable cause for tHPE. In contrast to the present study, no patients in the study by Lum et al. exhibited clinically overt HPP [16]. McKiernan et al. (2014) identified numerous conditions that may potentially cause tHPE, including anemia, major surgery, multisystem failure, tumors, sepsis, disseminated intravascular coagulopathy, gastrointestinal bleeding, severe caloric restriction, and blood transfusion [3]. It is important to note that these non-physiological conditions are usually reflected by the biomarkers investigated in the present study, as detailed in Table 2. Therefore, it is essential to consider these factors when assessing a decreased ALP serum activity level [3]. The evaluation of the AP, taking into account the general biomarkers, showed an accuracy of 90% and could discern between persistent HPE (ALPL group) and tHPE in 94% of the cases.

Furthermore, McKiernan et al. (2014) showed that tHPE is associated with a more severe decrease in ALP serum activity levels (to 10 U/L) and increased mortality rate compared to patients with HPP [3]. According to the literature, mean ALP serum activity levels in adult patients with confirmed HPP ranged from 27.8 U/L to 28.5 U/L, which is comparable to the mean ALP serum activity level of 22 U/L in the ALPL group studied [19]. However, it should be noted that a massively decreased ALP serum activity level may also indicate HPP due to homozygous PNVs in the *ALPL **gene*** or a childhood form diagnosed in adulthood [9]. It has also been suggested that bisphosphonate therapy may result in decreased ALP serum activity levels in up to 43% of patients [32]. Therefore, the significantly lower ALP serum activity levels in the tHPE group are consistent with data found in the literature and do not automatically imply HPP.

### 4.2. Offered Diagnostic Approach for a Suspected ALPL Gene Mutation

**Figure 2:**
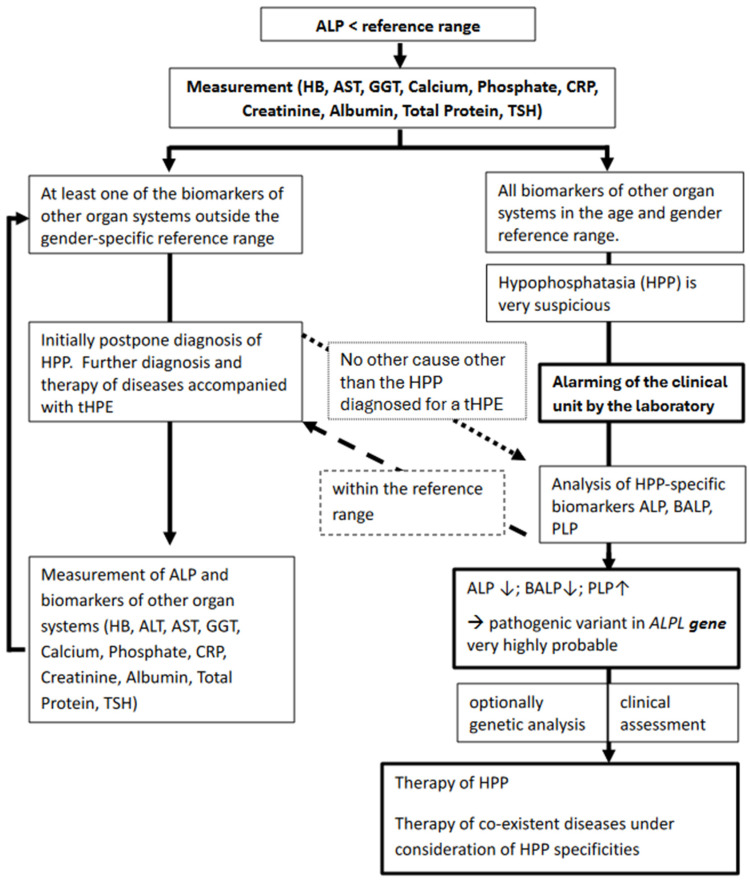
Overview of our recommended laboratory algorithm for the evaluation of a single low serum alkaline phosphatase (ALP) activity (hypophosphatasemia, HPE) result in the setting of a pathogenic nucleotide variant of the *ALPL **gene*** and possible differential diagnosis of temporary hypophosphatasemia (tHPE). ↓ = below the reference range; ↑ = above the reference range.

In the case of pHPE, HPP-specific diagnostics with determination of the BALP and its substrate PLP are usually recommended [17]. A decreased serum level in BALP and an increased level in its substrate PLP are biochemical findings that confirm HPP [17]. In summary, HPP-specific diagnostics are expensive, time-consuming, and not available universally. Considering the presented results, it can be argued that a reduction in alkaline phosphatase activity should be assessed in conjunction with a number of additional biomarkers. Of particular interest here are biomarkers that enable a differential diagnosis to be made. These include the levels of hemoglobin, aspartate aminotransferase, calcium, albumin, and total serum protein, as well as the levels of C-reactive protein (Figure 2).

First, in patients where a decreased ALP serum activity level has been identified alongside other general parameters within the reference range, a specific HPP diagnosis can be recommended. Second, in patients with one or more general parameters in the pathological range in addition to decreased ALP serum activity level, further investigation of the treatable differential diagnoses (Table 1) is strongly recommended. Following the targeted treatment of the differential diagnoses, a remeasurement of the biomarkers is recommended. In the event that the general biomarkers normalize concurrently with persistently decreased ALP serum activity levels, a PNV of the *ALPL **gene*** remains a probable explanation. In both situations, it is recommended that an extended HPP-specific diagnostic work-up be performed, including BALP and PLP levels [33]. A diagnosis of HPP can be made in the presence of additional clinical symptoms that are characteristic of HPP. In rare cases, a PNV of the *ALPL **gene*** can coexist with pathological values of the general biomarkers presented. In such cases, according to the literature, genetic analysis is recommended. However, if the patient presents with clinical and laboratory findings consistent with a diagnosis of HPP, it is not recommended that genetic analysis be conducted as a routine procedure [5,34,35].

### 4.3. Limitations of the Study

It should be noted that this study is not without limitations. Primarily, the retrospective design of the study may have introduced discrepancies in the number of observations for the various parameters, potentially affecting the validity of our conclusions. The precise number of patients who had their alkaline phosphatase levels measured at the UHL during the specified period could not be ascertained. During the inclusion period, software modifications were implemented, creating challenges in accessing historical data. The present study identified 10,464 cases in which at least one determination of ALP serum activity level was performed. Of these, 217 exhibited an ALP serum activity level of ≤21 U/L (0.2%). This information would have been interesting to estimate the frequency of low levels and how often they were ignored. Another drawback, despite the significant differences, was the number of patients who agreed to be followed up. While this was still relatively high in the tHPT group (88), only 11 patients in the ALPL group were available for complete data collection. Furthermore, the selected screening cut-off of 21 U/L for ALP serum activity level has the potential to result in an underdiagnosis of patients exhibiting a milder decrease in ALP serum activity levels due to PNVs of the *ALPL **gene***. The results suggest that the prevalence of moderate forms of genetic HPP in adults is likely to be higher than previously assumed, highlighting the necessity for greater attention to be paid to the serum activity level in ALP [10].

It should be noted that the presented algorithm cannot be applied unreservedly to forms of HPP resulting from homozygous or compound heterozygous PNVs in the *ALPL **gene***. The patients analyzed retrospectively in this study presented with an oligosymptomatic mild form of HPP associated with heterozygous pathogenic nucleotide variants *in the ALPL **gene***, which was consistent with the data in the literature [34,36,37]. Given the genetic heterogeneity of the disease, a genetically based nosology of HPP, as suggested by Mornet et al., may be a useful approach [34]. Nevertheless, the clinical relevance of such an entity also remains to be seen in the context of patient-specific, individualized therapy, which should be the subject of further investigation. The validity of the presented algorithm for this purpose must be corroborated by prospective studies, ideally including a comprehensive genetic diagnosis for tHPE.

## 5. Conclusions

The analysis of basic biomarkers has been demonstrated to facilitate the interpretation of decreased ALP serum activity levels in the context of potential HPP. In the event of a decreased ALP serum activity level, it would be advantageous if the basic biomarkers used could be automatically determined by the laboratory department. Moreover, it is advised that follow-up tests, including tests of the basic biomarkers, be conducted. In the event of a decreased ALP serum activity level and of basic biomarkers within the reference range, there is already a strong initial suspicion of a PNV in the *ALPL **gene***. In this situation, it is recommended that the clinical department be informed of the suspicion of HPP and that the laboratory department automatically determine the HPP-specific parameters. Otherwise, further diagnostics in addition to HPP are recommended in the presence of pathological values in basic biomarkers in the context of HPE.

## Figures and Tables

**Table 1:** Descriptive statistics and overview of diagnoses at the time of the inclusion of ALP serum activity level (lowest detected ALP serum activity level, *ALPL*; alkaline phosphatase *gene*; tHPE, temporary hypophosphatasemia, ^(1)^ chi-test 1.00, ^(2)^ *t*-test *p* = 0.286; ^(3)^ *t*-test *p* = 0.297; ^(4)^ U-test *p* = 0.931; * = 9.1; CCI, craniocerebral injury; ICH, intracranial hemorrhage.

	tHPE-Group (N = 88)		ALPL-Group (N = 11)	
Sex distribution ^(1)^	female: 46; male: 42		female: 6; male: 5	
mean age				
all ^(2)^	57.0 (SD 19.7)		51.6 (SD 14.7)	
female	54.9 (SD 20.4) ^(3)^		54.2 (SD 15.3) ^(4)^	
male	59.3 (SD 19.0) ^(3)^		48.4 (SD 15.0) ^(4)^	
main diagnosis at the time of determination of the inclusion of ALP	N	(%)	N	(%)
blood loss anemia				
gastrointestinal hemorrhage	2	2.3	2	18.2
traumatic acute hemorrhage	12	13.6		
postoperative hemorrhage	9	10.1		
cancer-induced chronic anemia	3	3.4		
acute myocardial ischemia	2	2.3		
neurological/neurosurgical diseases				
CCI without ICH	3	3.4		
CCI with ICH	1	1.1		
epileptic incident	2	2.3		
oligoastrocytoma	1	1.1		
cerebral stroke	4	4.5	1	*
optic nerve sheath meningioma			1	*
multiple system atrophy cerebellar variant	1	1.1		
systemic infections				
sepsis caused by various factors	9	9.1		
pneumonia	4	4.5		
gastrointestinal diseases				
liver insufficiency	9	10.3	1	*
acute abdomen by various diseases	5	5.4		
acute biliary pancreatitis	1	1.1		
colitis ulcerosa			1	*
hematological diseases				
multiple myeloma	6	6.5		
leukemia (several forms)	6	6.5		
non-Hodgkin’s lymphoma	2	2.3		
drug-induced agranulocytosis (cytostatica)	2	2.3		
rheumatological disease				
systemic lupus erythematosus (plasmapheresis)	2	2.3		
psoriatic arthritis				
arthralgias with unclear cause			2	18.2
ovarian hyperstimulation syndrome	1	1.1		
chorioretinitis	1	1.1		
acute renal failure			1	*
thoracal vertebral body fracture			1	*
dermatitis herpetiformis (Duhring)			1	*

**Table 2:** Differences in common biomarkers between patients with pathogenic nucleotide variants in the ALPL gene (ALPL group) and patients with temporary hypophosphatasemia (tHPE group).

	Group	N	Mean	SD	N (%) Within Reference Range	*p*
alkaline phosphatase (U/L)	ALPL	11	22.57	6.71	11 (100%)	0.005
	tHPE	88	15.25	5.49	88 (100%)	
aspartate amino-transferase (µkat/L)	ALPL	10	0.52	0.35	9 (90%)	0.004
	tHPE	71	1.85	3.68		
gamma-glutamyl transferase (µkat/L)	ALAP	10	1.60	2.78	7 (70%)	0.231
	tHPE	69	0.93	1.43	42 (61%)	
hemoglobin (mmol/L)	ALPL	11	8.46	0.85	10 (90%)	0.000
	tHPE	66	5.84	2.16	60 (91%)	
creatinine (µmol/L)	ALPL	9	70.33	36.52	8 (89%)	0.081
	tHPE	82	101.83	113.58	71 (86%)	
calcium (mmol/L)	ALPL	9	2.41	0.04	9 (100%)	0.001
	tHPE	62	1.73	0.61	49 (62%	
thyrotropin (mU/L)	ALPL	10	1.72	1.00	9 (90%)	0.159
	tHPE	34	1.17	1.23	29 (85%)	
C-reactive protein (mg/L)	ALPL	11	1.47	1.60	11 (100%)	0.039
	tHPE	66	39.27	59.50	59 (89%)	
albumin (g/L)	ALPL	11	46.05	3.06	11 (100%)	0.000
	tHPE	51	22.17	13.48	50 (98%)	
total protein (g/L)	ALPL	11	72.05	3.28	11 (100%)	0.000

**Table 3:** Overview of the pathological gene variants detected in the *ALPL **gene***, along with the corresponding measured follow-up AP, BAP, and PLP serum levels observed in patients with hypophosphatasia (HPP). F, female, M, male.

Pathogenic Nucleotide Variant in the *ALPL* Gene	ClinVar Variation ID	Frequency	Sex	Follow-Up-AP		BALP		PLP Level (Reverence Value, 35–110 nmol/L) nmol/L
				µkat/L	% of the Lower Reference Value	Reduced	Value µg/L	
c.119C > T; p.(Ala40Val) Exon 3	975919	1	M	0.41	61.2	yes	4.7	141
c.1250A > G; p.(Asn417Ser) Exon 11	13679	4	F M F M	0.25 0.23 0.32 0.43	43.1 34.2 55.2 64.2	yes yes yes no	3.1 4.3 4.5 6.7	144 241 198 308
c.1171del; p.(ARG391Valfs *12) Exon 10	632628	1	M	0.34	50.7	yes	4.6	112
c.1331A > G; p.(Gln444Arg) Exon 12	976385	1	M	0.26	38.8	yes	3.0	258
c.406C > T; p.(ARG136Cys) Exon 5	964572	1	F	0.45	77.6	no	5.9	100
c.341C > T; p.(Ala114Val) Exon 5	976384	1	W	0.52	89.7	no	5.4	128
c.1190-2A > T	976090	1	W	0.56	96.6	yes	3.8	366
c.297G > A; p.(=)	976088	1	W	0.31	53.5	yes	4.0	177

**Table 4:** Model comparison of the coefficients of ALPL and tHPE group.

	Accuracy	F1 Score	AUC
Model 1 ALP serum activity level	0.9	0.5	0.872549
Model 2 (all biomarkers)	0.9	0.666667	0.941176

**Table 5:** Coefficient of basic biomarkers from logistic regression.

Biomarker	Significance Between ALP and tHPE Groups	Coefficient
thyrotropin	0.159	0.900124
albumin	0.000	0.564765
gamma-glutamyl transferase	0.231	0.410748
total protein	0.000	0.243145
alkaline phosphatase	0.005	0.14744
creatinine	0.081	0.013057
calcium	0.001	−0.00362
aspartate amino-transferase	0.004	−0.17544
C-reactive protein	0.039	−0.67206
hemoglobin	0.000	−0.97785

## Data Availability

Please contact the corresponding authors in the case of any request for the data published here.
